# Mutational Analysis of a Highly Conserved PLSSMXP Sequence in the Small Subunit of *Bacillus licheniformis* γ-Glutamyltranspeptidase

**DOI:** 10.3390/biom9090508

**Published:** 2019-09-19

**Authors:** Meng-Chun Chi, Huei-Fen Lo, Min-Guan Lin, Yi-Yu Chen, Tzu-Fan Wang, Long-Liu Lin

**Affiliations:** 1 Department of Applied Chemistry, National Chiayi University, 300 Syuefu Road, Chiayi City 60004, Taiwan; s0910324@alumni.ncyu.edu.tw (M.-C.C.);; 2 Department of Food Science and Technology, Hungkuang University, 1018 Taiwan Boulevard, Shalu District, Taichung City 43302, Taiwan; hflo@sunrise.hk.edu.tw; 3 Institute of Molecular Biology, Academia Sinica, Nangang District, Taipei City 11529, Taiwan; am1988814@gate.sinica.edu.tw

**Keywords:** γ-Glutamyltranspeptidase, *Bacillus licheniformis*, self-activation, transpeptidation activity, deletion analysis, Ala-scanning mutagenesis

## Abstract

A highly conserved ^458^PLSSMXP^464^ sequence in the small subunit (S-subunit) of an industrially important *Bacillus licheniformis* γ-glutamyltranspeptidase (*Bl*GGT) was identified by sequence alignment. Molecular structures of the precursor mimic and the mature form of *Bl*GGT clearly reveal that this peptide sequence is in close spatial proximity to the self-processing and catalytic sites of the enzyme. To probe the role of this conserved sequence, ten mutant enzymes of *Bl*GGT were created through a series of deletion and alanine-scanning mutagenesis. SDS-PAGE and densitometric analyses showed that the intrinsic ability of *Bl*GGT to undergo autocatalytic processing was detrimentally affected by the deletion-associated mutations. However, loss of self-activating capacity was not obviously observed in most of the Ala-replacement mutants. The Ala-replacement mutants had a specific activity comparable to or greater than that of the wild-type enzyme; conversely, all deletion mutants completely lost their enzymatic activity. As compared with *Bl*GGT, S460A and S461S showed greatly enhanced *k*_cat_/*K*_m_ values by 2.73- and 2.67-fold, respectively. The intrinsic tryptophan fluorescence and circular dichroism spectral profiles of Ala-replacement and deletion mutants were typically similar to those of *Bl*GGT. However, heat and guanidine hydrochloride-induced unfolding transitions of the deletion-associated mutant proteins were severely reduced as compared with the wild-type enzyme. The predictive mutant models suggest that the microenvironments required for both self-activation and catalytic reaction of *Bl*GGT can be altered upon mutations.

## 1. Introduction

N-terminal nucleophile (Ntn) hydrolases are a group of evolutionarily-related enzymes that can hydrolyze the amide bonds in peptides and proteins [1]. Notably, enzymes within this superfamily become catalytically active after an intramolecular autoproteolysis of the precursor polypeptides [2,3,4,5,6]. The intramolecular processing of the inactive precursors is generally believed to proceed through the nucleophilic attack of oxygen or sulfur atom in the side chains of Thr, Ser, or Cys residues on the carbonyl group of the immediate upstream backbone to form a tetrahedral intermediate [5,6,7,8,9,10]. Following protonation of the amino group of the resulting intermediate and the subsequent N → O or N → S acyl shift, an ester is produced as an intermediate that is then hydrolyzed by an activated water molecule to yield the mature enzymes with Thr, Ser, or Cys as the N-terminal residue of the respective small subunit (S-subunit).

As a member of Ntn hydrolases, γ-glutamyltranspeptidase (GGT, EC 2.3.2.2) is capable of transferring the γ-glutamyl moiety from γ-glutamyl donors to many different types of amino acids and peptides or water [11]. It is widely distributed in all domains of life [12,13] and apparently involved in the regulation of the cellular ratio of reduced glutathione [14,15,16]. GGT enzymes are proposed to complete the reaction cycle through the following two consecutive steps (Scheme S1) [11]: (i) a general base-catalyzed nucleophilic attack on the amide bond of the substrates by the threonine hydroxyl group to form a transient acyl-enzyme adduct, and (ii) hydrolysis of the acyl-enzyme adduct to regenerate a catalytically competent enzyme. To date, the catalytic cycle of GGT enzymes is fully understood and requires a strictly conserved threonine residue [17], Thr391 in *Escherichia coli* GGT (*Ec*GGT), Thr381 in *Homo sapiens* GGT (*Hs*GGT), and Thr399 in *Bacillus licheniformis* GGT (*Bl*GGT), serving as a nucleophile to attack the carbonyl carbon of the γ-glutamyl substrates to yield a γ-glutamyl-enzyme adduct. Afterwards, the γ-glutamyl-enzyme adduct can react either with a water molecule to give glutamate in a hydrolysis reaction or with the γ-glutamyl acceptors to carry out a transpeptidation reaction.

To date, the crystal structures of numerous GGT enzymes, including *Ec*GGT [18], *Helicobacter pylori* GGT (*Hp*GGT) [6], *Bacillus subtilis* GGT (*Bs*GGT) [19], *Bl*GGT [20], and *Hs*GGT [21] have been determined. Similar to other members of Ntn hydrolases, the solved structures share a common overall architecture of four-layer αββα sandwich with two β-sheets packed against each other in an antiparallel orientation and sandwiched by layers of α-helices on either side. These crystal structures also allow us to understand that S-subunit and large subunit (L-subunit) are highly intertwined throughout the molecular architecture via both hydrogen bonds and hydrophobic contacts.

The maturation of GGT enzymes proceeds through a self-catalyzed intramolecular reaction that generates a catalytically active heterodimeric enzyme consisting of one L-subunit and one S-subunit [5,6,21]. The self-activation of GGT enzymes essentially follows the aforementioned mechanism of Ntn hydrolases [17]. As shown in Scheme S2, the hydroxyl group of the strictly conserved threonine (Thr399 in *Bl*GGT) of the proenzyme serves as a nucleophile for the cleavage. The base-activated Thr initially attacks the carbonyl group of the preceding residue (Glu398 in *Bl*GGT) to form a tetrahedral intermediate. The cleavage of the C–N bond through protonation of the amino group of the Thr yields an ester intermediate (N–O acyl shift), which is then hydrolyzed by a water molecule to produce two subunits. After the subunit assembly, the Thr nucleophile becomes the new N-terminal residue of the S-subunit of the heterodimeric active enzyme. In *Ec*GGT, *Hp*GGT, and *Bl*GGT [6,22,23], replacement of the evolutionarily conserved Thr residue (Thr391 in *Ec*GGT, Thr380 in *Hp*GGT, and Thr399 in *Bl*GGT) by Ala creates the mutant proteins that are unable to undergo the autoproteolytic activation and consequently leads to a detrimental impact on the catalytic activity. Through the experimental data collected from GGT enzymes of several organisms and the Ala-replacement mutants of *Bl*GGT, an advanced self-activating mechanism has been proposed [24]. In the revised mechanism [24], the main-chain carbonyl group of a Glu residue (Glu398 in *Bl*GGT) and the side-chain hydroxyl groups of two Thr residues (Thr399 and Thr417 in *Bl*GGT) are pertinent to the formation of a six-membered transition state.

**Figure 1:**
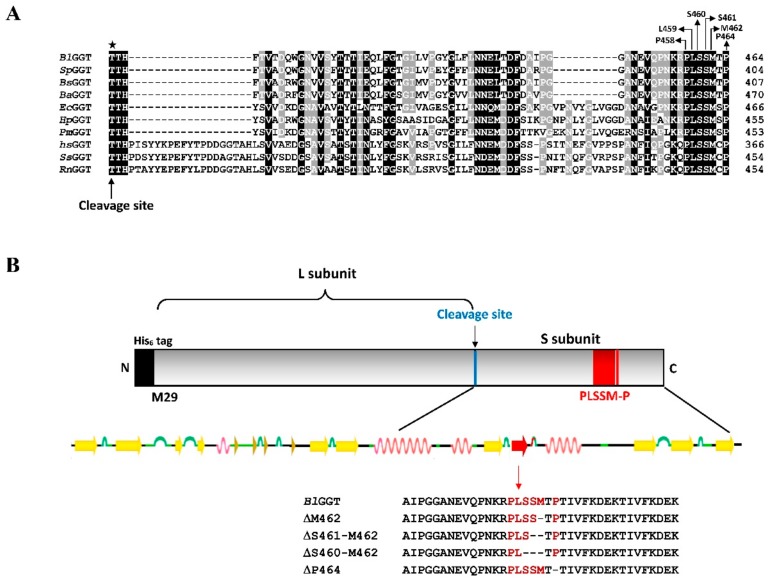
Multiple sequence alignment of γ-glutamyltranspeptidases (GGTs) from different organisms and schematic representation of deletion constructs. (**A**) The amino acid sequence of *Bl*GGT (UniProtKB-Q62WE3) is aligned with those of *Streptococcus pneumoniae* UniProtKB-A0A0U0D9D2), *B. subtilis* (UniProtKB-P54422), *Bacillus amyloliquefaciens* (UniProtKB-F4ELE6), *E. coli* (UniProtKB-P18956), *H. pylori* (UniProtKB-O25743), *Proteus mirabilis* (UniProtKB-B4EUO9), *H. sapiens* (UniProtKB-P19440), *Sus scrofa* (UniProtKB-P20735), and *Rattus norvegicus* (UniProtKB-P07314). The conserved catalytic Thr responsible for the self-activation of the enzyme is marked with a star. (**B**) Schematic diagram of the recombinant *Bl*GGT and the particular sequences of its deletion mutants.

Our previous works have already demonstrated that the C-terminal truncation and extra-sequence deletion of *Bl*GGT definitely affect the ability of the enzyme precursor to self-activate [25,26]. Actually, more new insights into the structure-function relationships in *Bl*GGT are of significant practical interest since the active form of the enzyme is being implicated in the biocatalytic synthesis of several naturally occurring γ-glutamyl compounds [27,28,29]. Through the use of the program CLUSTLAW from ExPASy Proteomics server (http://tw.expasy.org) to perform a multiple sequence alignment, we identified an evolutionarily conserved PLSSMXP sequence (residues 458–464 in *Bl*GGT and X stands for any amino acid) in the S-subunit of the aligned enzymes (Figure 1). The situation of these residues near the essential environment for the self-activation of *Bl*GGT and in the vicinity of the active cleft warrants their exploration as the critical residues for the enzyme (Figure S1). In the current study, an attempt has been made to exploit the role of this conserved sequence by deletion and Ala-scanning mutagenesis. The data presented herein suggest that the conserved ^458^PLSSMXP^464^ sequence may indirectly be involved in the functionality of *Bl*GGT, most likely through hydrogen-bonding and hydrophobic interactions with certain key amino acid residues to maintain the microenvironments required for the self-activation and catalysis.

## 2. Materials and Methods

### 2.1. Deletion and Ala-Scanning Mutagenesis

The recombinant plasmid pQE-*Bl*GGT, previously constructed in our laboratory [30], was used as the DNA template for polymerase chain reaction (PCR) based mutations. Deletion and Ala-scanning mutagenesis were carried out with a commercially available site-directed mutagenesis kit (QuikChange XL-II; Agilent Technologies, Santa Clara, CA, USA). The complementary mutagenic primer pairs for protein engineering were designed by ourselves (Table S1) and subjected to synthesis service (Mission Biotech Co., Ltd., Taipei, Taiwan). Reaction conditions for the PCR-based mutagenesis were essentially according to the suppliers’ instructions. Following amplification, *Dpn*I-treated PCR amplicons were transformed into XL-1-Blue supercompetent cells. Plasmid DNA isolated from the individual colonies of each transformation was sequenced to confirm the desired mutation. The mutated plasmids were accordingly called pQE-*Bl*GGT/ΔM462, pQE-*Bl*GGT/ΔS460-M462, pQE-*Bl*GGT/ΔS461-M462, pQE-*Bl*GGT/ΔP464, pQE-*Bl*GGT/P458A, pQE-*Bl*GGT/L459A, pQE-*Bl*GGT/S460A, pQE-*Bl*GGT/S461A, pQE-*Bl*GGT/M462A, and pQE-*Bl*GGT/P464A, respectively.

### 2.2. Protein Expression and Purification

Wild-type enzyme and its deletion and Ala-replacement mutants were all expressed in *E. coli* M15 (pREP4), released from the recombinant cells by sonication, and purified by nickel-chelate chromatography, as described previously [31]. The imidazole elution fractions were pooled and resolved by 12% sodium dodecyl sulfate (SDS) polyacrylamide gels to assess the purity of each preparation. Bradford assays were routinely performed with the ready-to-use protein assay dye (Bio-Rad) and the protein concentration of each preparation was determined by comparing the assay response to a standard curve of bovine serum albumin.

A straightforward approach to calculate the level of self-activation was accomplished by quantifying the densitometry of each protein band in the 12% SDS-polyacrylamide gels using a computerized densitometer coupled with the GelAnalyzer 2010a software (http://www.gelanalyzer.com). The processing rate of each enzyme can be estimated by dividing the combined densitometric volume of L- and S-subunits by the total densitometric volume of the precursor and processed bands. Each value represents the mean of three independent determinations.

### 2.3. Enzyme Assays

The transpeptidation activity of *Bl*GGT and its deletion and Ala-replacement mutants was measured by a colorimetric method as described elsewhere [32]. In a typical assay, the reaction mixture (0.5 mL) consisted of 25 mM Tris-HCl buffer (pH 9.0), 1.25 mM l-γ-glutamyl-*p*-nitroanilide (l-γ-Glu-*p*-NA), an excess of acceptor substrate Gly-Gly (30 mM), 1 mM MgCl_2_, and 0.1 mL of suitably diluted enzyme preparation (~6.0 μg/mL). After 10 min of reaction at either 40 °C for all enzymes, or 25 °C just only for the deletion mutants, a UV-visible spectrophotometer was employed to detect the released *p*-nitroaniline (*p*-NA) by measuring the absorbance at 410 nm. A standard curve of *p*-NA was established to determine the transpeptidation activity. One unit of the enzymatic activity is defined as the amount of enzymes that can liberate 1 μmol of *p*-NA from the chromogenic substrate per minute through the transpeptidation reaction. Data represent the mean of three independent assays.

Kinetic parameters of the wild-type and mutant enzymes were determined essentially as described previously [31]. To estimate the kinetic constants, a Lineweaver–Burk plot was established with data points derived from double-reciprocal transformation. Data represent the mean of three independent assays.

### 2.4. Spectroscopic Analyses

Fluorescence spectroscopic analysis of all protein samples was carried out at room temperature with a JASCO FP-6500 Spectrofluorometer (JASCO international Co., LTD., Tokyo, Japan). The excitation wavelength for the fluorescence spectroscopic analysis was set at 295 nm with a 1 nm bandwidth and the emission spectra of all protein samples were collected from 310 to 450 nm under a bandwidth of 10 nm. Prior to the spectroscopic study, all protein samples were diluted to approximately 12.5 μM with 25 mM Tris-HCl buffer (pH 9.0). Data acquisition and analysis were performed with JASCO’s unique cross platform spectroscopy software supplied by the manufacturer. Each fluorescence experiment was done independently five times.

Circular dichroism (CD) spectra of all protein samples in the far-ultraviolet region were acquired from a JASCO-815 Spectrophotopolarimeter (JASCO international Co., LTD., Tokyo, Japan) with a 0.1 cm optical path cuvette and the spectral data were recorded over a wavelength range of 250 to 190 nm at room temperature. A scanning speed of 20 nm/min was carried out with an averaging time of 4 s and a wavelength step of 0.2 nm. Signal averaging over 10 scans or more were used to record and the data for each spectrum was acquired independently three times. Prior to the CD study, all protein samples were diluted to approximately 24.5 μM in 25 mM Tris-HCl buffer (pH 9.0). The CD spectra were corrected with the control curve of 25 mM Tris-HCl buffer (pH 9.0). The molar residue ellipticity (MRE) of the analyzed samples can be calculated by the following formula: [*θ*] = *θ*_obs_ (in mdeg)/(the molar protein concentration × path length (in nm) × the total number of amino acid residues in the protein). The unit of molar residue ellipticity is deg · cm^2^ · dmol^−1^.

Thermal unfolding of all protein samples (~24.5 μM) in 25 mM Tris-HCl buffer (pH 9.0) was evaluated by monitoring the spectral change at 222 nm wavelength. Protein samples were heated from 20 to 90 °C with a constant heating rate of 1 °C/min. Melting transition temperature (*T*_m_) was calculated as described previously [33].

### 2.5. Chemical Denaturation

The chemical stability of *Bl*GGT and its deletion mutants was investigated by monitoring the changes in their fluorescence emission maximum of tryptophan as a function of guanidine hydrochloride (GdnHCl) concentrations. To obtain denaturation profiles, samples (1 mL) with increasing GdnHCl concentrations (0–2.5 M) were prepared by mixing 100 μL of enzyme preparation (~162 μM) with appropriate amounts of denaturant stock solution (3 M). The enzyme/GdnHCl mixtures were preincubated at room temperature for 30 min to allow for equilibration before spectroscopic analysis. Fluorescence emission spectra was determined by a JASCO FP-6500 Spectrofluorometer with an excitation wavelength of 295 nm and fluorescence emission was recorded from 310 to 500 nm. Both maximum wavelength shift and fluorescence intensity change were analyzed together to compute the average emission wavelength (AEW) [34].

### 2.6. Computer Modeling

Predictive models of the deletion and Ala-replacement mutants were fabricated by the Swiss-Model Server [35] using the three-dimensional structures of T399A-*Bl*GGT (PDB code: 4Y23) [24] and *Bl*GGT complexed with L-glutamate (PDB code: 4OTU) [20] as the templates. The mutant models were established by initially replacing or deleting the pertinent amino acids in the template structures and subsequently performing local side-chain minimization within 8 Å Cα-Cα distance from any residue of the interaction partner. Afterwards, the predictive models were subjected to an energy minimization (200 steps of steepest descent) with a partial implementation of the GROMOS force field accessible via the Swiss-pdb Viewer software v4.1.0 (http://spbdv.vital-it.ch/).

## 3. Results and Discussion

### 3.1. Local Environments Surrounding the Conserved ^458^PLSSMXP^464^ Sequence

In the molecular architecture of T399A-*Bl*GGT [24], a precursor mimic of *Bl*GGT, the side chain hydroxyl group of Thr417 is located within a competent position to act as a base to selectively deprotonate the OH group of Thr399 for an intramolecular nucleophilic attack, and the backbone atoms of this residue are held individually in their spatial positions by the positively charged guanidino group of Arg571 that, in turn, engages in a strong electrostatic-based interaction with the side-chain carboxyl group of Glu398. In addition, the correct spatial positions of the side-chain atoms of Thr399 are critical for the self-activation of *Bl*GGT and this positioning is assured by the hydrophobic interactions among nonpolar groups of Thr415 and Met462 [24]. Giving the fact that the proper spatial position of Thr417 is linked to a hydrogen-bonding network involving one sodium ion, two water molecules, and amino acid residues Pro458, Ser460, and Ser461 (Figure S1A), the amino acid residues within the conserved ^458^PLSSMXP^464^ sequence are likely to be involved in the autocatalytic processing of *Bl*GGT.

With the support of evidence-based research, many of the amino acid residues critical for the transpeptidation activity of GGT enzymes were being implicated [36,37,38,39,40,41,42,43], and the identification of key functional residues in the active site of *Bl*GGT has just been accomplished a few years ago by solving the crystal structure of this heterodimeric enzyme in complex with L-glutamate [20]. Notably, the substrate-binding site is deeply buried within a pocket that is lined by amino acid residues Arg109, Thr399, Thr417, Glu419, Glu438, Ser460, Ser461, Gly481, and Gly482 (Figure S1B). Over the past years, several researchers were interested in exploring the role of these residues in *Bl*GGT and the corresponding residues in its counterparts through the use of site-directed mutagenesis technique [23,36,38,41,42,43,44,45]. The study of Ikeda and his coworkers has identified two serine residues, Ser451 and Ser452 (Ser460 and Ser461 in *Bl*GGT), required for the catalysis of *Hs*GGT [38]. Two previous investigations relevant to *Ec*GGT have helped us further understand the functional role of Ser463 (Ser461 in *Bl*GGT) and Met464 (Met462 in *Bl*GGT) [41,43]. As shown in Figure 1A, the ^458^PLSSMXP^464^ sequence is highly conservative among GGT enzymes from a variety of species so that the amino acid residues within this peptide sequence may, therefore, play a role in the catalysis of *Bl*GGT.

### 3.2. Self-Activation and Catalytic Activity of the Wild-Type Enzyme and its Mutants

**Figure 2:**
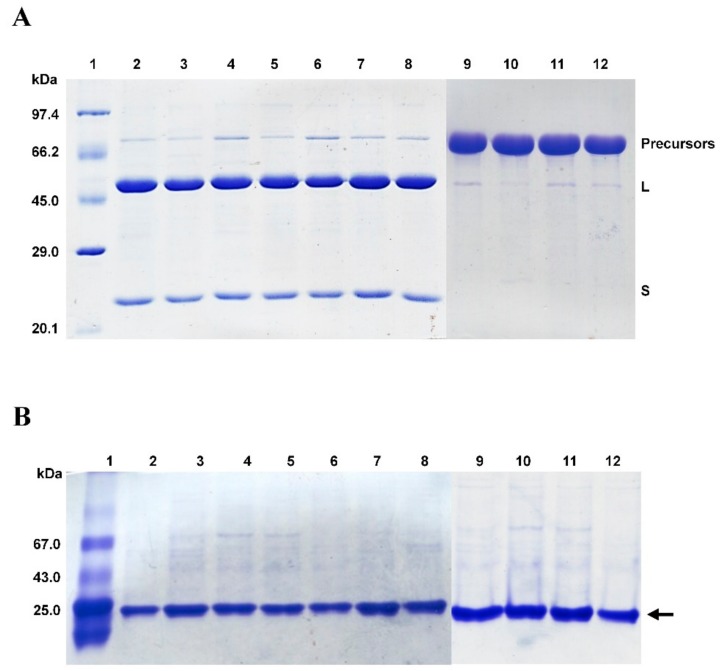
Polyacrylamide gel electrophoresis of the freshly prepared wild-type and mutant enzymes. (**A**) A representative analysis of the purified enzymes by 12% SDS-PAGE. Each lane was loaded with approximately 1.2 μg of protein. The gels were stained with Coomassie Brilliant Blue R-250 solution and destained in a solution of 30% (*v/v*) methanol and 10% (*v/v*) acetic acid. Lanes 1–12 denote protein molecular weight marker, *Bl*GGT, P458A, L459A, S460A, S461A, M462A, P464A, ΔM462, ΔS460-M462, ΔS461-M462, and ΔP464, respectively. (**B**) Native gel electrophoresis of the purified enzymes. Each lane was loaded with approximately 0.6 μg of protein and electrophoresis was performed in 1× TG buffer (GMbiolab Co. Ltd., Taipei, Taiwan) at room temperature for 40 min using a constant voltage of 100 V. Gels were stained and destained as described above. The arrow indicates the position of major band. Albumin (67 kDa), ovalbumin (43 kDa), and chymotrypsinogen A (25 kDa) were used as reference standards.

Site-directed mutagenesis techniques are one of the principal tools of molecular biology to study the sequence-structure-function relationships of a protein [46]. To probe the role of the amino acids within the conserved ^458^PLSSMXP^464^ sequence of *Bl*GGT, four deletion (ΔM462, ΔS461-M462, ΔS460-M462, and ΔP464) and six Ala-replacement (P458A, L459A, S460A, S461A, M462A, and P464A) mutants were generated by site-directed mutagenesis. The verified plasmids pQE-*Bl*GGT/ΔM462, pQE-*Bl*GGT/ΔS460-M462, pQE-*Bl*GGT/ΔS461-M462, pQE-*Bl*GGT/ΔP464, pQE-*Bl*GGT/P458A, pQE-*Bl*GGT/L459A, pQE-*Bl*GGT/S460A, pQE-*Bl*GGT/S461A, pQE-*Bl*GGT/M462A, and pQE-*Bl*GGT/P464A were separately transformed into the competent *E. coli* M15 (pREP4) cells by calcium chloride (CaCl_2_) heat shock method. Following recombinant gene expression in host cells and single-step protein purification, the freshly prepared enzyme samples were subjected to purity and molecular size analysis by 12% SDS-PAGE. As shown in Figure 2A, three protein bands with the individual molecular masses of about 72, 50, and 22 kDa were found in the freshly prepared *Bl*GGT sample. These bands were consistently present in Ala-replacement mutant preparations, while the precursor form was predominantly observed in the deletion-related mutant samples (Figure 2A). The purified wild-type and mutant proteins were also subjected to native gel electrophoresis. As shown in Figure 2B, just a single major band was observed in all enzyme preparations, indicating that the *Bl*GGT integrity remains unchanged after the mutations.

The extent of autocatalytic processing of each enzyme preparation was subsequently quantified by densitometry analysis of the protein bands on an SDS-PAGE gel stained with Coomassie Brilliant Blue R-250. The results of such analysis revealed that the freshly prepared *Bl*GGT was able to achieve a processing rate of 95.5% and three Ala-replacement mutant enzymes (L459A, S462A, and P464A) displayed a slight impairment in the capability to self-process (Table 1), with 4.5% to 13.1% reduction in the processing rate. Based on these observations, it can be concluded that the individual residues within the conserved ^458^PLSSMXP^464^ sequence are nevertheless absolutely essential for the autocatalytic processing of *Bl*GGT. Consistently, site-specific replacements of Asn450, Gly481, and Gly482 by other amino acids have shown only a certain degree of influence on the autocatalytic processing of *Bl*GGT [31,44]. Minor contribution of the aforementioned residues to the self-activation of *Bl*GGT may be due solely to the facts that they are just in close spatial proximity to the self-activating site and do not play a direct role in the autocatalytic processing of the enzyme. However, the deletion-associated mutations had a serious impact on the self-activation with processing rates of less than 8.3% (Table 1). This result indicates that detrimental changes in the microenvironment required for the autocatalytic processing of *Bl*GGT may probably occur as a result of the deletion mutations.

To verify whether the existing functionality was working as expected, the GGT activity of freshly prepared *Bl*GGT, P458A, L459A, S460A, S461A, M462A, P464A, ΔM462, ΔS460-M462, ΔS461-M462, and ΔP464 was accordingly determined. As shown in Table 1, the freshly prepared *Bl*GGT, P458A, L459A, M462A, and P464A had a specific activity of 14.4, 17.8, 14.6, 19.5, and 6.7 U/mg, respectively. The specific activity of S460A and S461A was significantly increased by 1.1- and 1.4-fold with respect to that of the wild-type enzyme. In contrast, single substitutions at residues Ser451 and Ser452 (Ser460 and Ser461 in *Bl*GGT) of *Hs*GGT with Ala yielded the mutant enzymes with only about 1% activity of the wild-type enzyme [38]. Our results seem to contradict the findings of Ikeda et al. [38] so that further experimental clarification is required prior to make a good conclusion.

Steady-state kinetic parameters of *Bl*GGT and its Ala-replacement mutants were determined by a series of measurements of the initial rate of the transpeptidation reaction. As shown in Table 2, most of the Ala-replacement mutants displayed *K*_m_-values close to that of the wild-type enzyme. P458A had an apparent *k*_cat_ value of 16.0 s^−1^, which resembles the turnover number (16.6 s^−1^) of *Bl*GGT. The apparent *k*_cat_ values of S460A, S461A, and M462A showed an increase to a certain extent, while L459A and P464A had more than 18% reduction. Notably S460A, S461A, and M462A exhibited 273%, 267%, and 51% higher catalytic efficiency (*k*_cat_/*K*_m_) over *Bl*GGT, respectively.

**Figure 3:**
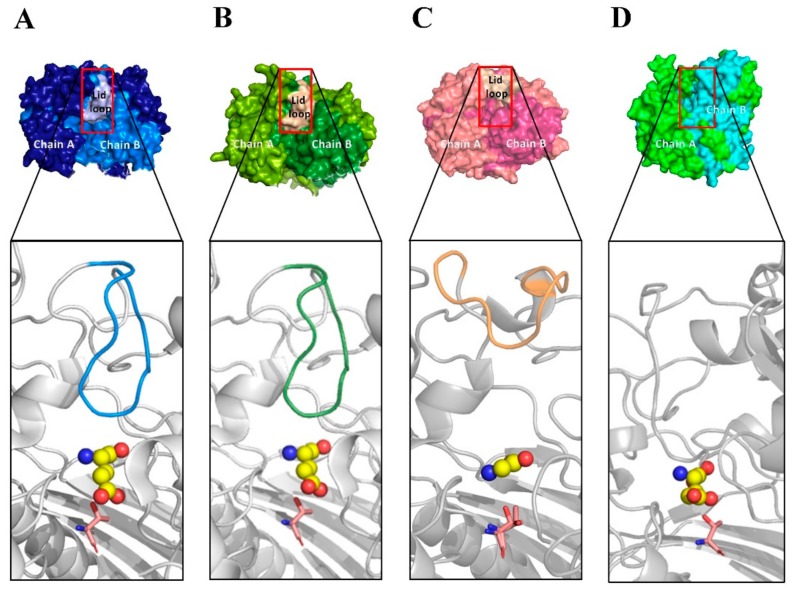
Comparison of the molecular surface and lip loop over the enzymatic pocket of four different GGT enzymes. The lid loop is colored in blue, green, and yellow; the catalytic threonine is shown as pink sticks; and the enzyme-bound L-glutamate is shown as spheres. Panels: (**A**) *Ec*GGT (2DBX), (**B**) *Hp*GGT (2QM6), (**C**) *Hs*GGT (4GDX), and (**D**) *Bl*GGT (4OTU).

**Figure 4:**
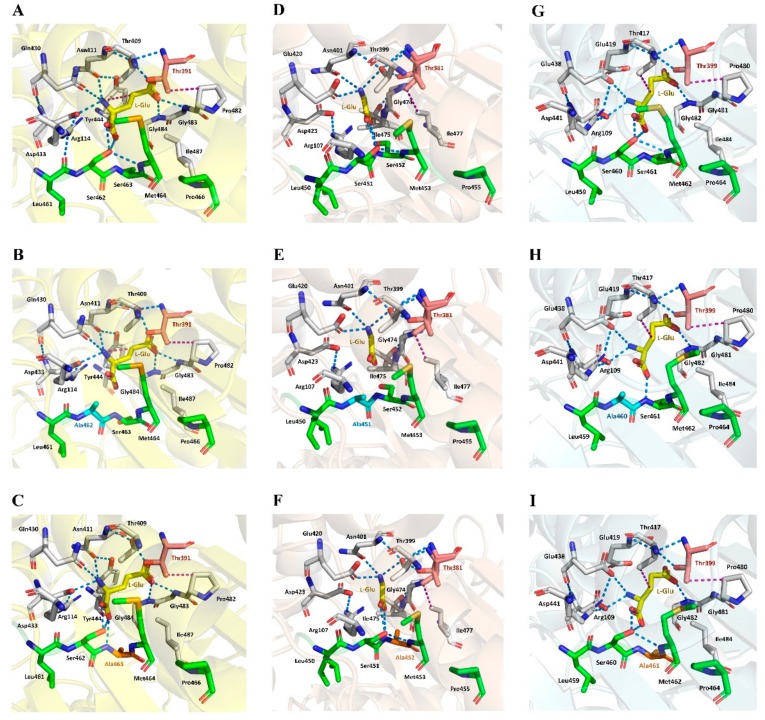
Enzyme-L-glutamate interactions within the substrate-binding pockets of *Ec*GGT, *Hs*GGT, and *Bl*GGT. The crystal structures of *Ec*GGT (2DBX) and *Hs*GGT (4GDX) were individually used as the templates to fabricate the predictive models of S462A-*Ec*GGT, S463A-*Ec*GGT, S451A-*Hs*GGT, and S452A-*Hs*GGT. Hydrogen-bonding and hydrophobic interaction are indicated by blue and purple dotted lines, respectively. The carbon skeletons of the conserved PLSSMXP sequence, catalytic threonine, and enzyme-bound l-glutamate are shown in green, pink, and yellow colors, respectively. Panels: (**A**) *Ec*GGT [18], (**B**) S462A-*Ec*GGT, (**C**) S463A-*Ec*GGT, (**D**) *Hs*GGT [21], (**E**) S451A-*Hs*GGT, (**F**) S452A-*Hs*GGT, (**G**) *Bl*GGT [20], (**H**) S460A, and (**I**) S461A.

Although all previously solved structures of GGT enzymes share a common overall architecture of four-layer αββα sandwich, they do not have similar primary structures. For example, the amino acid sequence of *Bl*GGT only exhibits 38.6% and 31.9% identity with those of *Ec*GGT and *Hs*GGT, respectively. In the crystal structures of *Ec*GGT and *Hp*GGT [18,47], there is a “lip loop” (Pro438-Gly449 in *Ec*GGT and Pro427 to Gly438 in *Hp*GGT) that covers most of the γ-glutamyl binding portion of the active site and that may block or limit the binding of acceptor molecules (Figure 3A,B). The molecular architecture of *Hs*GGT also shows that the anchoring termini of the lid loop are situated in comparable positions relative to those of *Ec*GGT and *Hp*GGT (Figure 3C), with its lip loop rotated away from the active site [21]. It has been documented by Castellano and Merlino [48] that the lid loop has a well-defined position and shields the enzymatic pocket from the solvent upon the binding site of a substrate or inhibitor. However, the analogous residues forming the lip loop are absent in *Bl*GGT [20], leading to the formation of a more open substrate channel in the active site (Figure 3D). Notably, the side chain of an exposed tyrosine (Tyr444 in *Ec*GGT and Tyr433 in *Hp*GGT) at the apex of the bacterial lid loop gates the active site by hydrogen bonding to a conserved asparagine residue (Asn411 in *Ec*GGT and Asn400 in *Hp*GGT) adjacent to the catalytic threonine [18,45]. The corresponding residue for gating the active site of *Hs*GGT is Phe433 [21], while its side chain is not hydrogen bonded with the analogous residue, Asn401. In our case, the absence of lip loop in *Bl*GGT apparently gives the enzymatic pocket more freedom for substrate entrance and product exit (Figure 3D). As shown in Table 2, the apparent *K*_m_ value of S460A was only 27% higher that of the wild-type enzyme and that for S461A was a little bit lower. These observations indicated that the enzyme-substrate affinity has not changed much after the replacements of Ser460 and Ser461 by alanine. The crystal structure of *Ec*GGT complexed with L-glutamate has further revealed that the carboxyl group is hydrogen bonded with Arg114 Nη, Ser462 Oγ, Ser463 N, and Ser463 Oγ via water, and the α-amino group has interactions with Asn411 Oδ, Gln430 Oε, and Asp433 Oδ (Figure 4A). The γ-glutamyl carbonyl oxygen of enzyme-bound L-glutamate is also hydrogen bonded with the main-chain amino atoms of Gly483 and Gly484. Besides, there are some hydrophobic interactions between hydrophobic parts in the ligand and the enzyme. It can be seen that a number of hydrogen-bonding interactions in the active site of *Ec*GGT are disrupted after the replacements of Ser462 and Ser463 by alanine (Figure 4B,C and Table S2). Interestingly, the enzyme-ligand interactions of *Ec*GGT are not perfectly conserved in the active sites of *Hs*GGT and *Bl*GGT (Figure 4D,G and Tables S3 and S4). As shown in Figure 4E,F and Table S3, Ala-replacement at Ser451 of *Hs*GGT has a certain degree of impact on the enzyme-ligand interactions, while the replacement of Ser452 by Ala does not make any changes on the original interactions. It is important to note that the hydrogen-bonding interactions within the enzymatic pocket of *Bl*GGT are almost entirely conserved in the predictive S460A and S461A models (Figure 4H,I and Table S4). Conclusively, a slight difference in the active-site architecture between *Bl*GGT and the compared enzymes together with the high conservation of the enzyme-ligand interactions may potentially explain why S460A and S461A can fully reserve their transpeptidation activity, whereas the respective mutants in *Ec*GGT and *Hs*GGT do not.

It is also worthy of mention that, under the standard assay conditions, there was no detectable transpeptidase activity in ΔM462, ΔS460-M462, ΔS461-M462, and ΔP464 (Table 1). This was further confirmed by performing the enzyme assay at 25 °C, indicating that the loss of their transpeptidation activity is unrelated to the heat-induced denaturation. Together with the above-mentioned results, it might be suggested that this conserved sequence is essential for the proper activity of *Bl*GGT and may indirectly involve in the reaction cycle by leaving the catalytic threonine (Thr399) and substrate in the spatial positions that efficiently facilitate nucleophilic attack.

### 3.3. Spectroscopic Characterization of the Structural Properties of Enzyme Preparations

**Figure 5:**
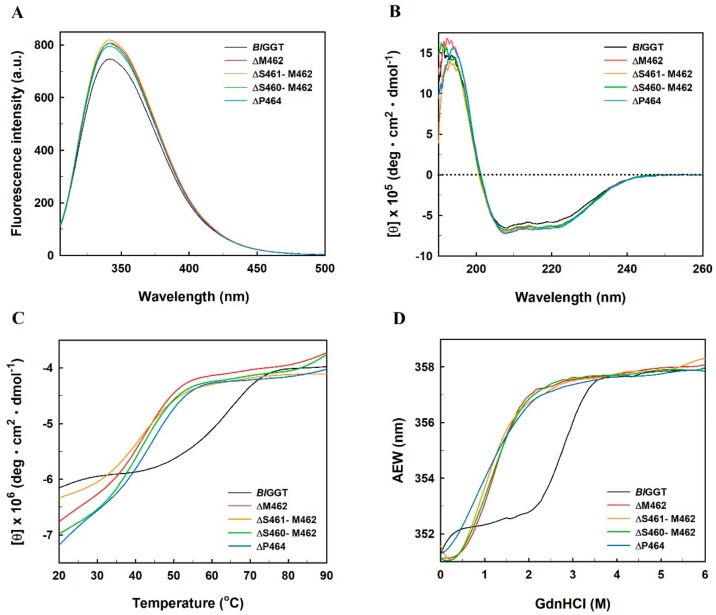
Spectroscopic analysis of the wild-type and deletion mutant enzymes. (**A**) Intrinsic fluorescence spectra of *Bl*GGT and its deletion mutants. The average of five spectra for each of the freshly prepared enzyme samples was recorded. (**B**) Far-UV spectra of *Bl*GGT and its deletion mutants. The data were recorded at 22 °C and residual molar elliticities of *Bl*GGT and its deletion mutants in 25 mM Tris-HCl buffer (pH 9.0) were measured from 190 to 250 nm. (**C**) Temperature-induced denaturation of *Bl*GGT and its deletion mutants. The protein samples in 25 mM Tris-HCl buffer (pH 9.0) were monitored with the CD signal at 222 nm. (**D**) GdnHCl-induced denaturation of *Bl*GGT and its deletion mutants. GdnHCl-induced alterations in the tertiary structures of the freshly prepared enzyme samples were monitored by calculating their AEWs.

Giving the fact that the structural impact caused by the studied mutations may contribute to changes in the enzymatic activity of *Bl*GGT, we here sought to investigate the structural alterations occurring in the deletion and Ala-replacement mutants by fluorescence and CD spectroscopy. As compared to the intrinsic tryptophan emission spectrum of *Bl*GGT, the fluorescence intensity of the freshly prepared deletion mutants was slightly enhanced by 6.5% to 9.7% (Figure 5A). It can be seen that the fluorescence spectra for *Bl*GGT and ΔMet462 were all maximized at a wavelength of 341.6 nm. Apparently, the fluorescence peak maxima of ΔS460-Met462, ΔS461-Met462, and ΔP464 were slightly shifted to either red or blue by less than 0.4 nm (Figure 5A). However, the freshly prepared Ala-replacement mutants exhibited up to 24% decrease in the fluorescence intensity, with no profound shift in the peak maxima (Figure S2A). These results indicate that only minor alterations in the molecular structure have occurred upon the introduction of deletion and Ala-replacement mutations into the conserved ^458^PLSSMXP^464^ sequence. Furthermore, there were no major differences in the CD spectra of wild-type enzyme and its relevant mutants (Figure 5B and Figure S2B). These results suggest that both deletion and Ala-replacement mutations have no detrimental consequences on the secondary structural content of *Bl*GGT. Conclusively, based on the results of fluorescence and CD measurements, we may exclude any gross structural changes for a conceivable explanation on the impaired activities of the mutant enzymes.

It is worthy of note that the extent of autocatalytic processing of the wild-type and mutant enzymes was not closely linked to the overall spectra of fluorescence and CD spectroscopy significantly (Figure 5A,B and Figure S2). Very recently, the crystal structure of T399A-*Bl*GGT has been solved at 2.89 Å resolution [24]. A Cα-carbon trace superposition of T399A-*Bl*GGT and the processed enzyme by Pica and his coworkers has shown that the root mean square error of the Cα atomic coordinates is 0.57 Å [24], which suggests a high degree of structural similarity between them. Therefore, the content of secondary and tertiary structural elements of each mutant appears to most closely resemble that of *Bl*GGT. This might be one possible explanation for why their CD and fluorescence spectra are highly similar to each other.

The irreversible inactivation of *Bl*GGT by heat has already been reported to follow the one-state process [49]. As shown in Figure 5C, the wild-type enzyme started to denature at around 48.1 °C and was transformed from its native state into a completely unfolded polypeptide chain at 70.2 °C. However, the unfolding process of the deletion mutants occurred at lower temperatures and ended up at 60.2 °C. The unfolding curves of *Bl*GGT, ΔM462, ΔS460-M462, ΔS461-M462, and ΔP464 all indicated one phase transition with an apparent *T*_m_ value of 61.8 ± 0.5, 42.1 ± 0.2, 41.2 ± 0.2, 41.9 ± 0.3, and 42.5 ± 1.2 °C, respectively. A review article has stated that there are numerous factors, including hydrogen-bonding networks, specific side-chain hydrophobic and packing interactions, and charge clusters, related to the thermal stability of proteins [50]. The results of heat-induced unfolding transition may give clues to the abolishment of some *Bl*GGT-stabilized forces by the deletion-associated mutations. In contrast, the apparent *T*_m_ values for S460A and S461A were relatively invariant at around 61 °C (Figure S2C). These results indicate that there is no significant change in the overall thermal stability of the enzyme as the consequences of Ser460 →Ala and Ser461 → Ala mutations.

Fluorescence spectroscopy is a sensitive, rapid and noninvasive analytical technique that can provide information on the conformational changes that underlie protein function [34]. The AEW that reports the changes of emission maximum and fluorescence intensity was used to assess the conformational stability of GdnHCl-treated enzymes. As shown in Figure 5D, the AEW value of *Bl*GGT in the absence and presence of 4.0 M GdnHCl was 351.2 and 357.3 nm, respectively. It has previously been stated that a fully exposed tryptophan residue in the completely unfolded protein has an emission maximum of approximately 356 nm [34]. In this regard, *Bl*GGT is completely unfolded under the GdnHCl concentrations of above 4 M. Alongside with that, the enzyme gave a midpoint transition of 2.9 M GdnHCl. However, the value of ΔM462, ΔS460-M462, ΔS461-M462, and ΔP464 was significantly reduced to 1.2, 1.2, 1.1, and 0.9 M, respectively. These observations clearly indicate that the structural integrity of *Bl*GGT are markedly affected by the deletion-associated mutations.

### 3.4. Impact of Mutations on the Spatial Positions of Self-Activating and Catalytic Residues

**Figure 6:**
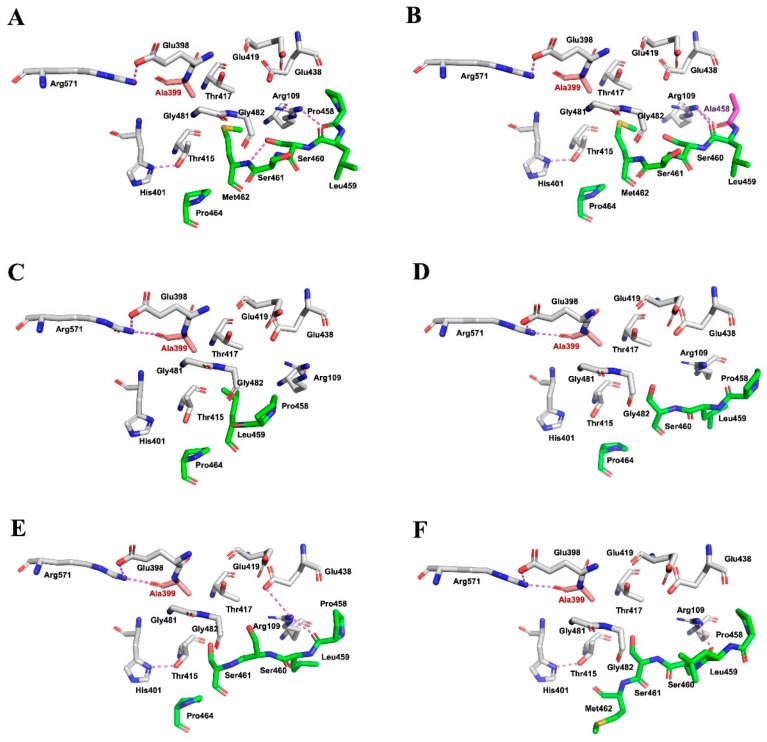
Comparison of the self-activating environments of wild-type and mutant enzymes. The self-activating environments were plotted by the program PyMOL (https://pymol.org). Important residues, including Arg109, Glu398, Ala399, His401, Thr415, Thr417, Glu419, Glu438, Gly481, Gly482, and Arg571, are shown. The self-activating environments of wild-type and mutant enzymes were individually presented in panels (**A)** (*Bl*GGT), (**B**) (P458A), (**C)** (ΔS460-M462), (**D)** (ΔS461-M462), (**E**) (ΔM462), and (**F**) (ΔP464).

Detailed knowledge about the three-dimensional structure of enzymes is crucial for studying their structure-function relationship. X-ray and NMR are the most frequently used experimental methods for solving enzyme structures, but this would be a much more difficult and time-consuming task during protein structure determination [51]. Thus, computer-based modeling provides an attractive option to generate predictive models of three-dimensional enzyme structures [52]. Computer modeling of the local environment surrounding the conserved ^458^PLSSMXP^464^ sequence was, therefore, performed to elucidate the impact of the deletion-associated mutations on the self-activation of *Bl*GGT. As it was mentioned above, amino acid residues Glu398, Thr415, Thr417, Arg571, and Met462 may play a practical role during the autocatalytic processing of *Bl*GGT (Figure S1A and Figure 6A). The likely spatial positions of these residues in the predictive model of P458A basically resemble those of the wild-type enzyme (Figure 6B). The conservation of the spatial locations of these residues allows this mutant enzyme to ensure an efficient self-activation (Table 1). It is worthy of note that the spatial arrangements of Glu398, Thr415, Thr417, and Arg571 are slightly altered in the predictive models of ΔS460-M462, ΔS461-M462, and ΔM462 (Figure 6C–E). Except for the above changes, a more profound shift in the spatial arrangement of Met462 can be seen in the model of ΔP464 (Figure 6F). Such changes in the spatial arrangements appear to interfere with the hydrogen-bonding networks and hydrophobic interactions that primarily held these critical residues in their relative spatial positions of *Bl*GGT (Figure S1A).

**Figure 7:**
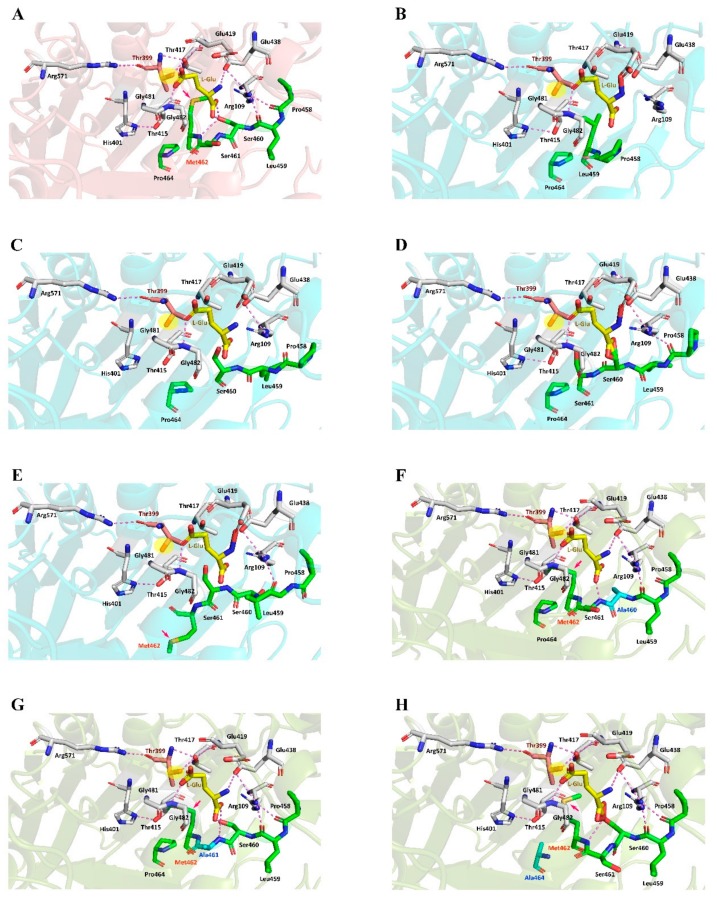
Comparison of the catalytic environments of wild-type and mutant enzymes. The catalytic environments were plotted by the program PyMOL (https://pymol.org). Critical residues, including Arg109, Thr399, His401, Thr415, Thr417, Glu419, Glu438, Gly481, Gly482, and Arg571, are shown. The catalytic environments of wild-type and mutant enzymes were individually presented in panels (**A**) (*Bl*GGT), (**B**) (ΔS460-M462), (**C**) (ΔS461-M462), (**D**) (ΔM462), (**E**) (ΔP464), (**F**) (S460A), (**G**) (S461A), and (**H**) (P464A). The carbon skeleton of the PLSSMXP sequence is highlighted in green and the hydrogen bonds are indicated by pink dashed lines. The side-chain hydroxyl group of the catalytic Thr399 is shaded in yellow and the side-chain position of Met462 is indicated by a pink arrow.

The crystal structure of *Bl*GGT complexed with an l-glutamate molecule has recently been determined and refined to an atomic resolution of 3.022 Å [20]. This study suggests that some important amino-acid residues, including Arg109, Glu438, Ser460, Ser461, Gly481, and Gly482, might be involved in l-glutamate binding. As shown in Figure S3, the predictive models of P458A, L459A, and M462A display small differences in the spatial positioning of L-glutamate-binding residues. This could be the reason why these three mutants exhibited the specific transpeptidation activity comparable to that of the wild-type enzyme. In the catalytic cycle of *Bl*GGT, the spatial orientation of the side-chain hydroxyl group of the catalytic Thr399 is critical for the nucleophilic attack at the carbonyl carbon of the γ-glutamyl substrates (Figure 7A and Scheme S1). As shown in Figure 7B–E, the spatial orientation of this side-chain hydroxyl group is dramatically altered upon different combinations of sequence deletion. In the most widely accepted reaction mechanism (Scheme S1), the side-chain hydroxyl group of the highly conserved Thr (Thr399 in *Bl*GGT), which is also responsible for the autocatalytic processing, attacks the C = O group of the γ-glutamyl-compound to form a γ-glutamyl-enzyme intermediate. The intermediate then reacts with water, to release glutamate in a hydrolysis reaction, or with an acceptor, to give a transpeptidation reaction forming new γ-glutamyl compounds. Therefore, alterations to the spatial orientation of this side-chain hydroxyl group will definitely affect the nucleophilic attack and eventually lead to the full abolishment of the enzymatic activity (Table 1).

It is also worthy of mention that the spatial position of Met462 in *Bl*GGT is notably changed after the site-directed replacement of Ser460 and Ser461 with Ala (Figure 7F,G). These spatial rearrangements will in turn change the hydrogen-bonding networks and open up the catalytic site of the enzyme, which may be more favorable to the entrance of the substrates and finally improve the catalytic activity of S460A and S461A. Although the spatial position of Met462 is notably shifted in the predictive models of S460A and S461A, the spatial orientation of the side-chain hydroxyl group of the catalytic Thr399 remains unchanged (Figure 7F,G). The spatial reservation of the catalytic Thr399 is probably the key that allows these two enzymes to work properly. As noted earlier, P464A only retained about 47% of the specific transpeptidation activity as compared to the wild-type enzyme. The profound decrease in the specific transpeptidation activity is probably caused by a slight change in the spatial positioning of Met462 after the Ala-replacement (Figure 7H).

## 4. Conclusions

In summary, the results of the combined effort of mutational and functional investigations rationalize the significance of the conserved ^458^PLSSMXP^464^ sequence in the autocatalytic processing and enzymatic activity of *Bl*GGT. Clearly, the secondary and tertiary structures of *Bl*GGT have not been changed profoundly by the deletion and Ala-replacement mutations, but the spatial alterations in amino acid residues involved in autocatalytic processing and L-glutamate-binding have really happened to some of the mutants. In the deletion-associated mutants, the disruption of hydrogen-bonding networks in the local surrounding environment of the active site may freeze the nucleophilic residue (Thr399) in a catalytically incompetent state that in turn leads to a complete loss of the enzymatic activity. However, further work is needed to more precisely elucidate the functional influence of Ala-replacements, especially the structural determinations of S460A and S461A.

## Figures and Tables

**Table 1:** The processing rate and specific activity of the freshly prepared wild-type and mutant enzymes.

Enzyme	Processing Rate (%)	Specific Activity (U/mg)
*Bl*GGT	95.5 ± 3.4	14.4 ± 1.9
P458A	98.0 ± 4.8	17.8 ± 2.1
L459A	86.0 ± 5.1	14.6 ± 1.2
S461A	95.8 ± 3.1	30.4 ± 2.4
S462A	83.0 ± 7.2	34.1 ± 2.0
M462A	93.0 ± 5.0	19.5 ± 1.0
P464A	91.2 ± 2.3	6.7 ± 0.3
ΔM462	8.3 ± 0.7	ND^a^
ΔS460-M462	6.3 ± 0.3	ND
ΔS461-M462	8.1 ± 0.4	ND
ΔP464	5.2 ± 0.6	ND

^a^ ND, not detected.

**Table 2:** Kinetic parameters of the freshly prepared wild-type and Ala-replacement enzymes.

Enzyme	*K*_m_ (μM)	*k*_cat_ (s^−1^)	*k*_cat_/*K*_m_ (s^−1^ μM^−1^)
*Bl*GGT	410.3 ± 5.4	16.6 ± 1.4	40.6 × 10^−3^
P458A	392.1 ± 1.9	16.0 ± 1.1	41.1 × 10^−3^
L459A	449.2 ± 4.7	13.6 ± 2.0	30.3 × 10^−3^
S460A	522.3 ± 6.5	78.8 ± 9.4	151.5 × 10^−3^
S461A	391.4 ± 4.3	58.1 ± 6.2	149.0 × 10^−3^
M462A	438.6 ± 5.2	26.8 ± 1.9	60.8 × 10^−3^
P464A	492.4 ± 7.4	8.7 ± 0.8	17.8 × 10^−3^

## Supplementary Materials

The following are available online at https://www.mdpi.com/2218-273X/9/9/508/s1, Figure S1: Local environments surrounding the highly conserved PLSSMXP region of *Bl*GGT, Figure S2: Intrinsic fluorescence (A) and Far-UV (B) spectra, and thermal unfolding curves (C) of *Bl*GGT, S460A, and S461A, Figure S3: The catalytic environments of P458A (A), L459A (B), and M462A (C), Table S1: Overlapping complementary primers used in the site-directed mutagenesis, Table S2: Comparison of hydrogen-bonding interactions in the enzymatic pockets of *Ec*GGT and the predictive S451A-*Ec*GGT and S452A-*Ec*GGT models, Table S3: Comparison of the hydrogen-bonding interactions in the enzymatic pockets of *Hs*GGT and the predictive S451A-*Hs*GGT and S452A-*Hs*GGT models, Table S4: Comparison of the hydrogen-bonding interactions in the enzymatic pockets of *Bl*GGT and the predictive S460A and S461A models, Scheme S1: The proposed catalytic mechanism of GGT enzymes, Scheme S2: The proposed mechanism for intramolecular autocatalytic processing of *Bl*GGT.

## References

[1] Oinonen C., Rouvinen J. (2000). Structural comparison of Ntn-hydrolases. Protein Sci..

[2] Brannigan J.A., Dodson G., Duggleby H.J., Moody P.C.E., Smith J.L., Tomchick D.R., Murzin A.G. (1995). A protein catalytic framework with an N-terminal nucleophile is capable of self-activation. Nature.

[3] Schmidtke G., Kraft R., Kostka S., Henklein P., Frömmel C., Löwe J., Huber R., Kloetzel P.M. (1996). Analysis of mammalian 20S proteasome biogenesis: The maturation of β-subunits is an ordered two-step mechanism involving autocatalysis. EMBO J..

[4] Guan C., Liu Y., Shao Y., Cui T., Liao W., Ewei A., Whitaker R., Paulus H. (1998). Characterization and functional analysis of the *cis*-autoproteolysis active center of glycosylasparaginase. J. Chem. Biol..

[5] Suzuki H., Kamagai H. (2002). Autocatalytic processing of γ-glutamylpeptidase. J. Biol. Chem..

[6] Boanca G., Sand A., Okada T., Suzuki H., Kumagai H., Fukuyama K., Barycki J.J. (2007). Autoprocessing of *Helicobacter pylori* γ-glutamyltranspeptidase leads to the formation of a threonine-threonine catalytic dyad. J. Biol. Chem..

[7] Ditzel L., Huber R., Mann K., Heinemeyer W., Wolf D.H., Groll M. (1998). Conformational constraints for protein self-cleavage in the proteasome. J. Mol. Biol..

[8] Xu Q., Buckley D., Guan C., Guo H.C. (1999). Structural insights into the mechanism of intramolecular proteolysis. Cell.

[9] Hewitt L., Kasche V., Lummer K., Lewis R.J., Murshudov G.N., Verma C.S., Dodson G.G., Wilson K.S. (2000). Structure of a slow processing precursor penicillin acylase from *Escherichia coli* reveals the linker peptide blocking the active-site cleft. J. Mol. Biol..

[10] Kim J.K., Yang I.S., Shin H.J., Cho K.J., Ryu E.K., Kim S.H., Park S.S., Kim K.H. (2006). Insight into autoproteolytic activation from the structure of cephalosporin acylase: A protein with proteolytic chemistries. Proc. Natl. Acad. Sci. USA.

[11] Keillor J.W., Castonguay R., Lherbet C. (2005). γ-Glutamyl transpeptidase substrate specificity and catalytic mechanism. Methods Enzymol..

[12] Tate S., Meister A. (1981). γ-Glutamyl transpeptidase: Catalytic, structural and functional aspects. Mol. Cell. Biochem..

[13] Chikhi N., Holic N., Guellaen G., Laperche Y. (1999). γ-Glutamyltranspeptidase gene organization and expression: A comparative analysis in rat, mouse, pig and human species. Comp. Biochem. Physiol. B Biochem. Mol. Biol..

[14] Jaspers C., Penniuckx M. (1984). Glutathione metabolism in *Saccharomyces cerevisiae*: Evidence that γ-glutamyltranspeptidase is a vacuolar enzyme. Biochimie.

[15] Suzuki H., Hashimoto W., Kumagai H. (1999). Glutathione metabolism in *Escherichia coli*. J. Mol. Catal. B Enzym..

[16] Hultberg M., Hultberg B. (2005). Glutathione turnover in human cell lines in the presence of agents with glutathione influencing potential with or with-out acivicin inhibition of γ-glutamyltranspeptidase. Biochim. Biophys. Acta.

[17] Castellano I., Merlino A. (2012). γ-Glutamyltranspeptidases: Sequence, structure, biochemical properties, and biotechnological applications. Cell. Mol. Life Sci..

[18] Okada T., Suzuki H., Wada K., Kumagai H., Fukuyama K. (2006). Crystal structures of γ-glutamyltranspeptidase from *Escherichia coli*, a key enzyme in glutathione metabolism, and its reaction intermediate. Proc. Natl. Acad. Sci. USA.

[19] Ida T., Suzuki H., Fukuyama K., Hiratake J., Wada K. (2014). Structure of *Bacillus subtilis* γ-glutamyltranspeptidase in complex with acivicin: Diversity of the binding mode of a classical and electrophilic active-site-directed glutamate analogue. Acta Crystallogr. D Biol. Crystallogr..

[20] Lin L.L., Chen Y.Y., Chi M.C., Merlino A. (2014). Low resolution X-ray structure of γ-glutamyltranspeptidase from *Bacillus licheniformis*: Opened active site cleft and a cluster of acid residues potentially involved in the recognition of a metal ion. Biochim. Biophys. Acta.

[21] West M.B., Chen Y., Wickham S., Heroux A., Cahill K., Hanigan M.H., Mooers B.H.M. (2013). Novel insights into eukaryotic γ-glutamyltranspeptidase 1 from the crystal structure of the glutamate-bound human enzyme. J. Biol. Chem..

[22] Okada T., Suzuki H., Wada K., Kumagai H., Fukuyama K. (2007). Crystal structure of the γ-glutamyltranspeptidase precursor protein from *Escherichia coli*. Structural changes upon autocatalytic processing and implications for the maturation mechanism. J. Biol. Chem..

[23] Lyu R.C., Hu H.Y., Kuo L.Y., Lo H.F., Ong P.L., Chang H.P., Lin L.L. (2009). Role of the conserved Thr399 and Thr417 residues of a recombinant *Bacillus licheniformis* γ-glutamyltranspeptidase as evaluated by mutational analysis. Curr. Microbiol..

[24] Pica A., Chi M.C., D’Ischia M., Chen Y.Y., Lin L.L., Merlino A. (2016). The maturation mechanism of γ-glutamyltranspeptidases: Insights from the crystal structure of a precursor mimic of the enzyme from *Bacillus licheniformis*. Biochim. Biophys. Acta.

[25] Chang H.P., Liang W.C., Lyu R.C., Chi M.C., Wang T.F., Su K.L., Hung H.C., Lin L.L. (2010). Effects of C-terminal truncation on the autocatalytic processing of a recombinant γ-glutamyltranspeptidase from *Bacillus licheniformis*. Biochemistry-Moscow.

[26] Chi M.C., Lo Y.H., Chen Y.Y., Lin L.L., Merlino A. (2014). γ-Glutamyl transpeptidase architecture: Effect of extra sequence deletion on autoprocessing, structure and stability of the protein from *Bacillus licheniformis*. Biochim. Biophys. Acta.

[27] Chen Y.Y., Lo H.F., Wang T.F., Lin M.G., Lin L.L., Chi M.C. (2015). Enzymatic synthesis of γ-L-glutamyl-*S*-allyl-L-cysteine, a naturally occurring organosulfur compound from garlic, by *Bacillus licheniformis* γ-glutamyltranspeptidase. Enzyme Microb. Technol..

[28] Chi M.C., Lo H.F., Lin M.G., Chen Y.Y., Lin L.L., Wang T.F. (2017). Application of *Bacillus licheniformis* γ-glutamyltranspeptidase to the biocatalytic synthesis of γ-glutamyl-phenylalanine. Biocatal. Agric. Biotechnol..

[29] Lee Y.C., Chi M.C., Lin M.G., Chen Y.Y., Lin L.L., Wang T.F. (2018). Biocatalytic synthesis of γ-glutamyl-L-leucine, a kokumi-imparting dipeptide, by *Bacillus licheniformis* γ-glutamyltranspeptidase. Food Biotechnol..

[30] Lin L.L., Chou P.R., Hua Y.W., Hsu W.H. (2006). Overexpression, one-step purification, and biochemical characterization of a recombinant γ-glutamyltranspeptidase from *Bacilllus licheniformis*. Appl. Microbiol. Biotechnol..

[31] Lin M.G., Chi M.C., Chen Y.Y., Wang T.F., Lo H.F., Lin L.L. (2016). Site-directed mutagenesis of a conserved Asn450 residue of *Bacillus licheniformis* γ-glutamyltranspeptidase. Int. J. Biol. Macromol..

[32] Tate S.S., Meister A. (1985). γ-Glutamyl transpeptidase from kidney. Methods Enzymol..

[33] Greenfield N.J. (2004). Analysis of circular dichroism data. Methods Enzymol..

[34] Lakowicz J.R. (2006). Principles of Fluorescence Spectroscopy.

[35] Arnold A., Bordoli L., Kopp J., Schwede T. (2006). The Swiss-Model workspace: A web-based environment for protein structure homology modelling. Bioinformatics.

[36] Ikeda Y., Fujii J., Taniguchi N. (1993). Significance of Arg-107 and Glu-108 in the catalytic mechanism of human γ-glutamyl transpeptidase. J. Biol. Chem..

[37] Ikeda Y., Fujii J., Taniguchi N., Meister A. (1995). Human γ-glutamyl transpeptidase mutants involving conserved aspartate residues and the unique cysteine residue of the light subunit. J. Biol. Chem..

[38] Ikeda Y., Fujii J., Anderson M.E., Taniguchi N., Meister A. (1995). Involvement of Ser-451 and Ser-452 in the catalysis of human γ-glutamyl transpeptidase. J. Biol. Chem..

[39] Ikeda Y., Fujii J., Taniguchi N. (1996). Effects of substitutions of the conserved histidine residues in human γ-glutamyl transpeptidase. J. Biochem. (Tokyo).

[40] Minami H., Suzuki H., Kumagai H. (2003). A mutant *Bacillus subtilis* γ-glutamyltranspeptidase specialized in hydrolysis activity. FEMS Microbiol. Lett..

[41] Lo H.F., Lin L.L., Chen P.J., Chou W.M. (2007). Site-directed mutagenesis of the conserved Thr407, Asp433, and Met464 residues in the small subunit of *Escherichia coli* γ-glutamyltranspeptidase. Indian J. Biochem. Biophys..

[42] Ong P.L., Yao Y.F., Weng Y.M., Hsu W.H., Lin L.L. (2008). Residues Arg114 and Arg337 are critical for the proper function of *Escherichia coli* γ-glutamyltranspeptidase. Biochem. Biophys. Res. Commun..

[43] Hsu W.H., Ong P.L., Chen S.C., Lin L.L. (2009). Contribution of Ser463 residue to the enzymatic and processing activities of *Escherichia coli* γ-glutamyltranspeptidase. Indian J. Biochem. Biophys..

[44] Chi M.C., Lin M.G., Chen Y.Y., Lin L.L., Wang T.F. (2018). Functional role of the conserved glycine residues, Gly481 and Gly482, of the γ-glutamyltranspeptidase from *Bacillus licheniformis*. Int. J. Biol. Macromol..

[45] Bindal S., Sharma S., Singh T.P., Gupta R. (2017). Evolving transpeptidase and hydrolytic variants of γ-glutamyltranspeptidase from *Bacillus licheniformis* by targeted mutations of conserved residue Arg109 and their biotechnological relevance. J. Biotechnol..

[46] Hsieh P.C., Vaisvila R. (2013). Protein engineering: Single or multiple site-directed mutagenesis. Methods Mol. Biol..

[47] Morrow A.L., Williams K., Sand A., Boanca G., Barycki J.J. (2007). Characterization of *Helicobacter pylori* γ-glutamyltranspeptidase reveals the molecular basis for substrate specificity and a critical role for the tyrosine 433-containing loop in catalysis. Biochemistry.

[48] Castellano I., Merlino A. (2013). γ-Glutamyl transpeptidases: Structure and function. SpringerBriefs in Biochemistry and Molecular Biology.

[49] Yang J.C., Liang W.C., Chen Y.Y., Chi M.C., Lo H.F., Chen H.L., Lin L.L. (2011). Biophysical characterization of *Bacillus licheniformis* and *Escherichia coli* γ-glutamyltranspeptidases: A comparative analysis. Int. J. Biol. Macromol..

[50] Jaenicke R., Bohm G. (1998). The stability of proteins in extreme environments. Curr. Opin. Struct. Biol..

[51] Krishnan V.V., Rupp B. (2006). Macromolecular Structure Determination: Comparison of X-ray Crystallography and NMR. eLS.

[52] Schwede T. (2013). Protein modeling: What happened to the “protein structure gap”?. Structure.

