# Supplementary material for: Mutational Analysis of a Highly Conserved PLSSMXP Sequence in the Small Subunit of Bacillus licheniformis γ-Glutamyltranspeptidase
Source: Biomolecules. 2019 Sep 19;9(9):508. doi: 10.3390/biom9090508 (PMC6769717; doi:10.3390/biom9090508)
Supplement: Supplementary file 1 [file biomolecules-09-00508-s001.pdf]

Supplementary materials

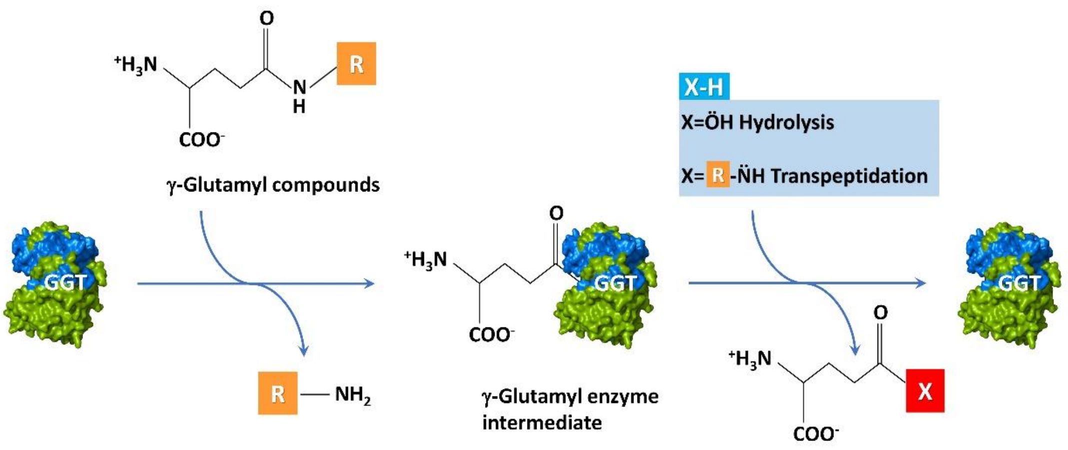

Scheme S1. The proposed catalytic mechanism of GGT enzymes.

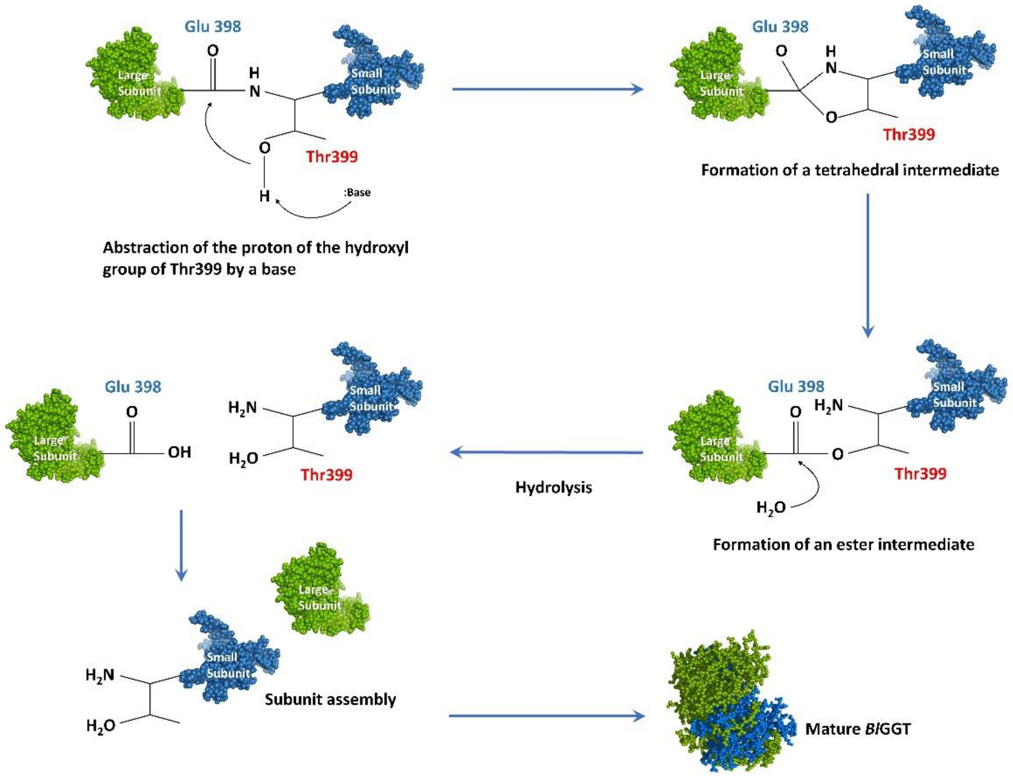

Scheme S2. The proposed mechanism for intramolecular autocatalytic processing of *Bl*GGT.

Table S1 Overlapping complementary primers used in the site-directed mutagenesis

Protein	Nucleotide sequence (5′→3′)	Codon change
△M462(f)	AAAAGGCCGCTTTCAAGCACGCCGACGATCGTATTC	-
△M462(r)	GAATACGATCGTCGGCGTGCTTGAAAGCGGCCTTTT	-
△S461-462(f)	AATAAAAGGCCGCTTTCAACGCCGACGATCGTATTC	-
△S461-462(r)	GAATACGATCGTCGGCGTTGAAAGCGGCCTTTTATT	-
△S460-462(f)	CCGAATAAAAGGCCGCTTACGCCGACGATCGTATTC	-
△S460-462(r)	GAATACGATCGTCGGCGTAAGCGGCCTTTTATTCGG	-
△P464(f)	CCGCTTTCAAGCATGACGACGATCGTATTCAAAGAT	-
△P464(r)	ATCTTTGAATACGATCGTCGTCATGCTTGAAAGCGG	-
P458A(f)	CCGAATAAAAGGGCGCTTTCAAGCATG	CCG→GCG
P458A(r)	CATGCTTGAAAGCGCCCTTTTATTCGG	
L459A(f)	AATAAAAGGCCGGCTTCAAGCATGACG	CTT→GCT
L459A(r)	CGTCATGCTTGAAGCCGGCCTTTTATT	
S460A(f)	AAAAGGCCGCTTGCAAGCATGACGCCG	TCA→GCA
S460A(r)	CGGCGTCATGCTTGCAAGCGGCCTTTT	
S461A(f)	AGGCCGCTTTCAGCCATGACGCCGACG	AGC→GCC
S461A(r)	CGTCGGCGTCATGGCTGAAAGCGGCCT	
M462A(f)	CCGCTTTCAAGCGCGACGCCGACGATC	ACG→GCG
M462A(r)	GATCGTCGGCGTCGCGCTTGAAAGCGG	
P464A(f)	TCAAGCATGACGGCGACGATCGTATTC	CCG→GCG
P464A(r)	GAATACGATCGTCGCCGTCATGCTTGA	

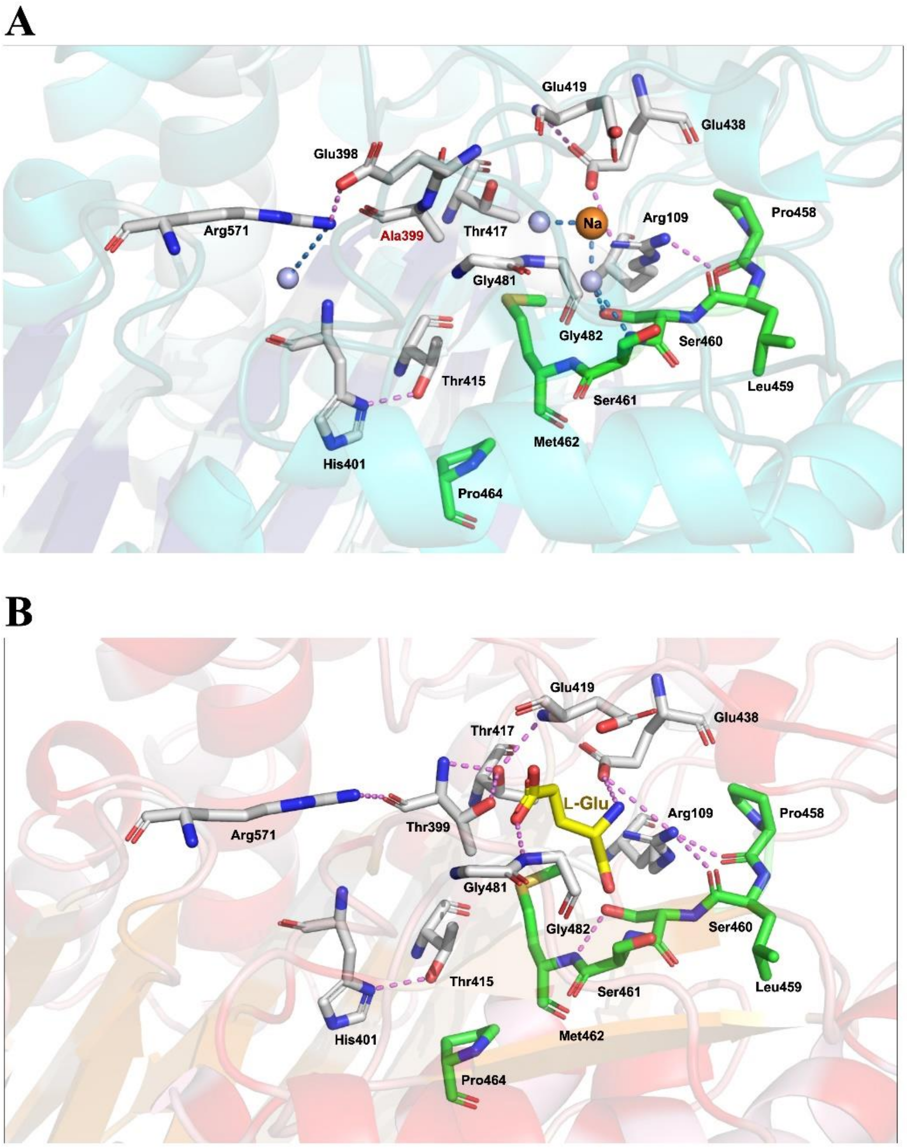

Fig. S1. Local environments surrounding the highly conserved PLSSMXP region of *Bl*GGT. (**A**) The self-activation environment of the T399A-*Bl*GGT structure (PDB 4Y23). The carbon skeleton of the PLSSMXP segment is highlighted in green color. The hydrogen bonds are indicated by pink dashed lines. (**B**) The interaction environment of the *Bl*GGT structure complexed with L-Glu (PDB 4OTU). The carbon skeleton of the PLSSMXP segment is highlighted in green color, whereas that of L-Glu is marked in yellow color. The hydrogen bonds are indicated by pink dashed lines.

Table S2. Comparison of hydrogen-bond interactions in the enzymatic pockets of *Ec*GGT and the predictive S451A-*Ec*GGT and S452A-*Ec*GGT models

Hydrogen bond	*Ec*GGT	S462A-*Ec*GGT	S463A-*Ec*GGT
Ligand…Asn411	√	√	√
Ligand…Gln430	√	√	√
Ligand…Asp433	√	√	√
Ligand…Ser462_1	√	×	√
Ligand…Ser462_2	√	×	√
Ligand…Ser463	√	×	√
Ligand…Gly483	√	√	√
Ligand…Gly484	√	√	√
Arg114…Asp433	√	√	√
Arg114…Leu461	√	×	×
Arg109…Glu438	√	√	√
Thr391…Thr409_1	√	√	√
Thr391…Thr409_2	√	√	√
Thr409…Asn411	√	√	√
Gln430…Asp433	√	√	×
Asn411…Tyr444	√	√	√
Ser462…Met464	√	×	×

Symbols: √, interaction; ×, no interaction.

Table S3. Comparison of the hydrogen-bond interactions in the enzymatic pockets of *Hs*GGT and the predictive S451A-*Hs*GGT and S452A-*Hs*GGT models

Hydrogen bond	*Ec*GGT	S451A-*Hs*GGT	S452A-*Hs*GGT
Ligand…Thr399	√	√	√
Ligand…Asn401	√	√	√
Ligand…Glu420	√	√	√
Ligand…Ser451_1	√	×	√
Ligand…Ser451_2	√	×	√
Arg107…Asp423	√	√	√
Thr381…Thr399_1	√	√	√
Thr381…Thr399_2	√	√	√
Thr381…Thr399_3	√	√	√
Ser451…Met453	√	√	√
Gln430…Asp433	√	×	√

Asn411…Tyr444	√	√	√
Gly474…Gly475	√	√	√

Symbols: √, interaction; ×, no interaction.

Table S4. Comparison of the hydrogen-bond interactions in the enzymatic pockets of *Bl*GGT and the predictive S460A and S461A models

Hydrogen Bond	*Bl*GGT	S460A	S461A
Ligand…Asp441	√	√	√
Ligand…Glu438	√	√	√
Ligand…Ser460_1	√	√	√
Ligand…Ser460_2	√	×	√
Ligand…Gly482	√	√	√
Arg109…Asp441	√	√	√
Arg109…Glu438	√	√	√
Thr399…Thr417_1	√	√	√
Thr399…Thr417_2	√	√	√
Thr417…Glu419	√	√	√
Glu438…Asp441	√	√	√
Ser460…Met462	√	√	√

Symbols: √, interaction; ×, no interaction.

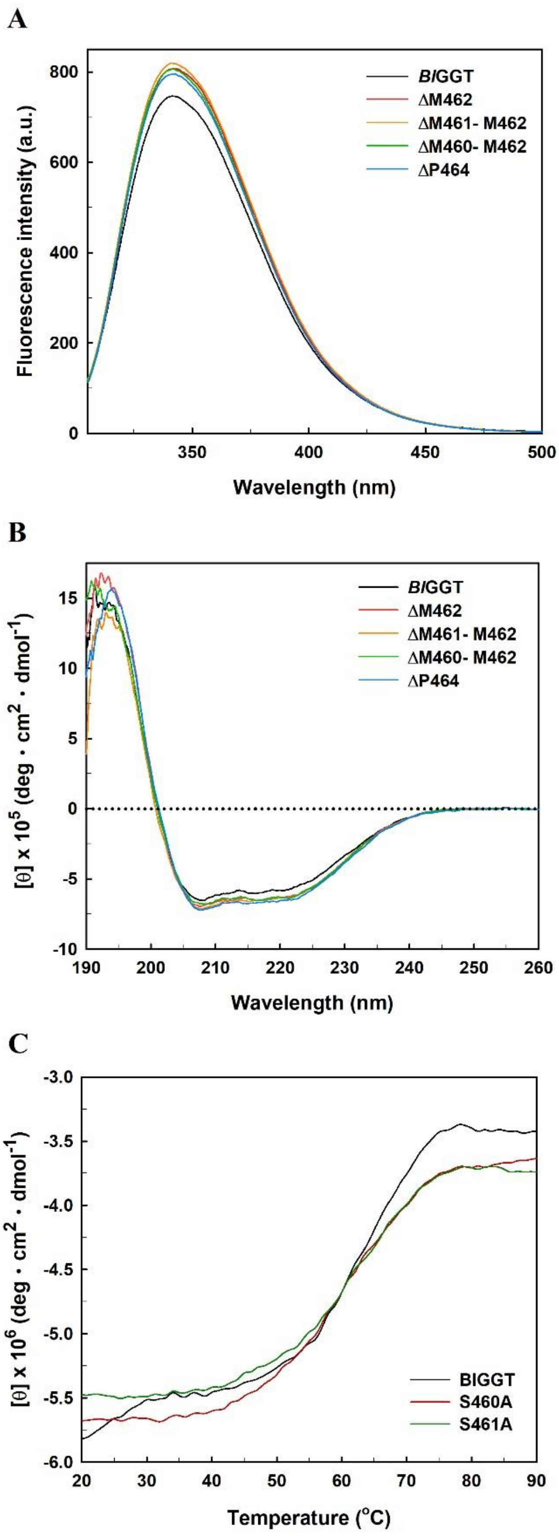

Fig. S2. Intrinsic fluorescence (**A**) and Far-UV (**B**) spectra, and thermal unfolding curves (**C**) of *Bl*GGT, S460A and S461A.

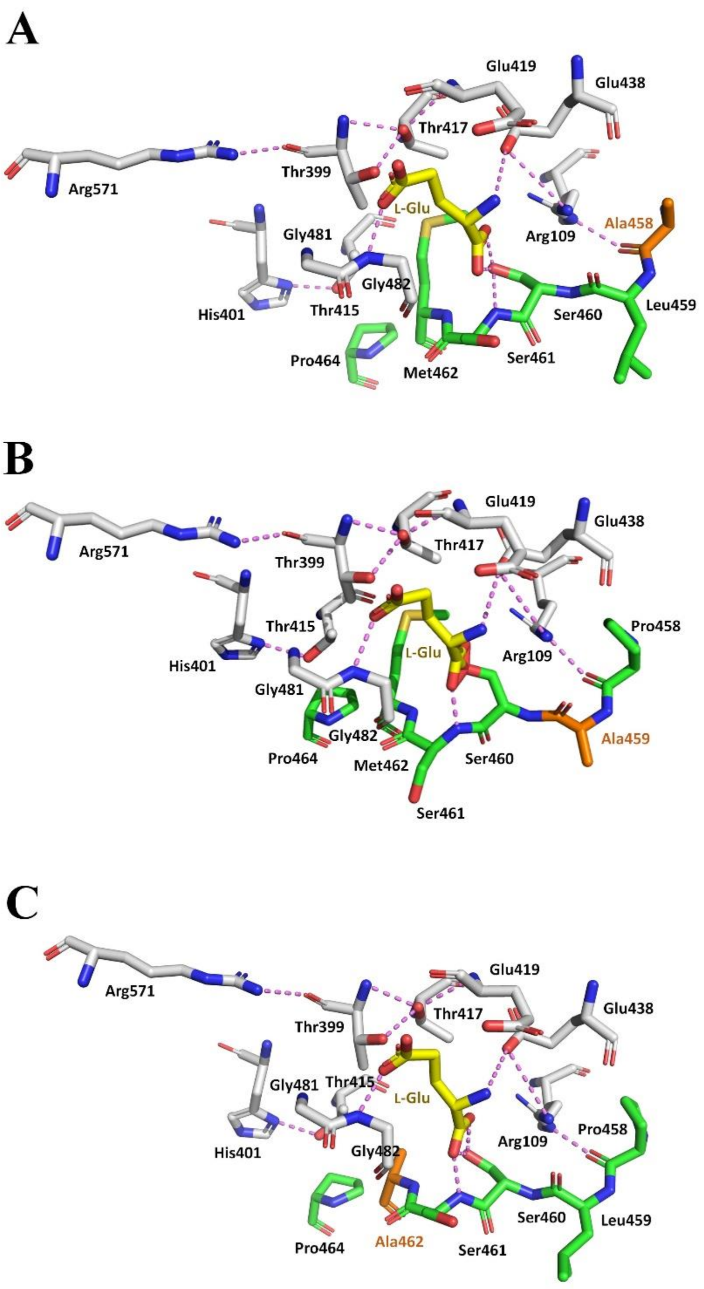

Fig. S3. The catalytic environments of P458A (A), L459A (B), and M462A (C). The catalytic environments were plotted by the program PyMOL (https://pymol.org). Critical residues Arg109, Thr399, His401, Thr415, Thr417, Glu419, Glu438, Gly481, Gly482, and Arg571 are shown. The carbon skeleton of the PLSSMXP sequence is highlighted in green color. The hydrogen bonds are indicated by pink dashed lines.
